# Stepping out of the shadows of Alzheimer’s disease: a phenomenological hermeneutic study of older people with Alzheimer’s disease caring for a therapy dog

**DOI:** 10.1080/17482631.2017.1347013

**Published:** 2017-07-12

**Authors:** Anna Swall, Britt Ebbeskog, Carina Lundh Hagelin, Ingegerd Fagerberg

**Affiliations:** ^a^ Department of Health Sciences, School of Health and Social Studies, Dalarna University, Falun, Sweden; ^b^ Karolinska Institutet, Department of Neurobiology, Care Science and Society, Stockholm, Sweden; ^c^ Sophiahemmet University, Department of Nursing Science, Stockholm, Sweden; ^d^ Department of Health Care Science, Ersta Sköndal Bräcke University College, Stockholm, Sweden

**Keywords:** Alzheimer’s disease, caring, animal-assisted therapy, person-centredness, phenomenological hermeneutics, life world

## Abstract

**Purpose**: Living with Alzheimer’s disease (AD) can involve a person being unable to recall and convey information in daily life. There are several ways to provide person-centred care to older people with AD, e.g. by empowering them in a situation. The use of animal-assisted therapy (AAT) with a therapy dog in the care of people with dementia is increasing, with the presence of a therapy dog being described as improving, among other things, the well-being and socialization of the person. The aim of this study was to illuminate meanings of care for people with AD in their encounters with a therapy dog.

**Method**: The study used video-recorded observations of the person with AD and the dog. Data were transcribed and analysed using a phenomenological hermeneutic method.

**Results**: The main theme was “Using one’s own resources and abilities as a human being”, which meant being the person one can be and distancing oneself from the symptoms of AD during the time with the dog.

**Conclusions**: The feelings evoked in the people with AD included empathy and altruism, which allowed for a sense of joy and tenderness, which may induce a sense of self-worth, of being needed, and of being meaningful.

## Introduction

A person-centred approach for people with Alzheimer’s disease (AD) and other dementias is beneficial when it comes to reaffirming and empowering the person (Edvardsson, Petersson, Sjogren, Lindkvist, & Sandman, 2013; Edvardsson, Winblad, & Sandman, 2008; Kitwood, 1997; McCormack & McCane, 2010). There are different methods for promoting person-centredness for people with dementia. A few examples are caregivers singing (Hammar, Emami, Götell, & Engström, 2011), validation therapy (Söderlund, Norberg, & Hansebo, 2012), and person-centred conversations (Hedman, Hansebo, Ternestedt, Hellström, & Norberg, 2012). These methods are often used as memory triggers, so-called reminiscence (Woods, Spector, Jones, Orrell, & Davies, 2005), and temporary presence of mind in the moment (Normann, Asplund, & Norberg, 1998).

Person-centred methods have been shown to strengthen the sense of “self” and identity among those who have dementia (Crichton & Koch, 2007; Hedman et al., 2012; Hedman, Hellström, Ternestedt, Hansebo, & Norberg, 2014). Loss of identity, or self, as a result of AD is a temporary loss of one’s self (Sabat, 2001), something Edvardsson et al. (2008) describe as being a dormant state of the person’s “personhood”, not a lost one. In addition, living with AD and other dementias can mean living one’s daily life in a nursing home in a state of uncertainty, confusion, and emotional instability, and being unable to recall or convey information (Sabat, 2001). Situations involving vulnerability and difficult symptoms are challenging to manage not only for someone with AD but also for the caregivers (Gates, Fitzwater, & Succop, 2003; Pulsford & Duxbury, 2006). Therefore, being involved in meaningful activities is important for the dignity and identity of people with dementia (Tranvåg, Petersen, & Nåden, 2013).

Throughout history, humans and animals have been of value to each other (Birke & Holmberg, 2011; Fine, 2010). In recent years, animals have been introduced into the care of people with dementia (Höök, 2010) and, according to Fine (2010), animals can serve as an extended arm for social interaction between humans. The presence of an animal can also help to facilitate contact between caregiver and patient. Animal-assisted therapy (AAT) with, for example, cats, fish, and dogs, is used in dementia care to counter emotional and psychological difficulties (Fine, 2010). AAT with a therapy dog has achieved positive results, such as increased social behaviour, improved quality of life (Bernabei et al., 2013; Marx et al., 2010), reduced aggression and anxiety (Kanamori et al., 2001; Mossello et al., 2011; Perkins Bartlett, Travers & Rand, 2008; Richeson, 2003; Sellers, 2005), and increased physical activity among people with dementia (Nordgren & Engström, 2012). AAT evokes memories, and there is a sense of physical closeness during the time the person spends stroking the dog. However, interaction with a therapy dog can also evoke sadness that may be difficult for people to handle (Swall et al., 2014). The involvement of a therapy dog in the care of people with dementia needs to be dealt with individually, and further studies are required (National Board of Health and Welfare, 2014).

To increase understanding of how the encounter with the therapy dog influenced the self of the person with AD, his or her verbal and non-verbal expressions of care for the dog were studied. This adheres to the definition of care given by Martinsen (1994), who defines the concept of caring as follows: “To care for” someone relates to “helping” and “taking care of” and to reducing the amount of “egoism” in each person, and is also related to the giving of oneself. The aim of this study, therefore, was to illuminate meanings of care for people with AD in their encounters with a therapy dog.

## Method

The lived experience of being in the world has its ontological and epistemological roots in the philosophies of Husserl (2004) and Ricouer (1976). What we experience in our daily lives is important for how we experience our life world. The present study aims to allow for a deeper understanding of the participants’ life world and the phenomenon studied, that being the lived experience of people with AD providing care for a dog. The life world of people with dementia may sometimes create for them a sense of vulnerability and fear, a result of the symptoms of the disease. In order to better understand the lived experience of old people who have AD, while highlighting the meanings of their lived experience when caring for a therapy dog, the phenomenological hermeneutic research method was used (Lindseth & Norberg, 2004).

### Therapy dog team

The therapy dog team comprises the handler and the handler’s own trained dog, which lives with the handler. To qualify as a therapy dog team in Sweden, the handler needs to have healthcare training—for example, as a registered nurse, nursing assistant, or occupational therapist—and the handler and dog must have completed an 18 month course at the Swedish therapy dog school. The dogs are trained to interact effectively in different situations with people with AD and other dementias. The dogs are thoroughly tested to ensure that they can handle different situations and perform well in interaction with older people with dementia. People with AD are prescribed visits from the therapy dog team (the dog and its handler) at the nursing home to cater for their different needs; for example, to decrease anxiety or improve their sense of well-being.

The sessions for each person took place once a week for a total of 10 weeks. The dog handler controlled the dog, talked to the person about the dog, and allowed the person to interact, while observing the situation the whole time. Sometimes the handler withdrew for a moment to give the person and the dog time alone, but was available to control the situation if needed. The therapy dog team visited those who had experiences with dogs earlier in their lives. The referrals were made by a registered nurse on the ward and individualized for each person with specific aims. Each session with the therapy dog was a one-on-one visit between the person with AD and the therapy dog team, and differed according to the circumstances of each person and the aim of the visit. The interaction between the person and the therapy dog occurred in a specially adapted room that was equipped with toys, blankets, pictures of dogs, chairs, and tables. There was also a couch where the person could sit down with the dog. Activities included close contact with the dog, talking, cuddling, and playing—such as throwing balls or searching for hidden sweets.

### Data collection

The study was conducted at a municipal nursing home in a metropolitan area in Sweden that comprised four inpatient wards for older people with dementia. Four women and one man between the ages of 89 and 95 years with medium to severe AD were included in the study. Inclusion criteria for those in the study at the specific nursing home were that they had been diagnosed with AD and that they had never received visits from a therapy dog team before. Those who did not like dogs or who did not have any previous relations to dogs were excluded in accordance with the decision of the dog handlers. The participants had all been prescribed a therapy dog team visit and met the inclusion criteria.

Data consisted of video observations (VIOs) aimed at capturing the behaviour of the people with AD within the context of their interaction with the therapy dog (Barker, Pistrang, & Elliott, 2005). Each visit was recorded (10 visits per person) (in total 50 sessions), and 25 h of video-recorded data were gathered by the first author. The observations focused on the interaction between the person with AD and the therapy dog, with the first author sitting in the room behind the camera filming the interaction. At the end of the visit, the first author asked the participants what they thought of the visit, and the question and the spoken answer, or perhaps no answer at all, were recorded on film. This enabled the person with AD to express in words his or her experience of interacting with the therapy dog.

### Data analysis

In the present study, the people with AD communicated about their life world when caring for a therapy dog through verbal and non-verbal communication (bodily movement, speech, and eye contact). The VIOs from the sessions were viewed and transcribed into text without interpretation (Swall et al., 2014). The transcribed texts were then separated from the VIOs and, according to Ricoeur (1976), the text then “stands on its own”. When an interpretation of the text is initiated using phenomenological hermeneutics, there is a circular movement between explanation and understanding, and between the parts and the whole of the text (the hermeneutic circle) (Lindseth & Norberg, 2004). The alternation between distance from the text and closeness to the text allows the researcher to capture what the text is about (Lindseth & Norberg, 2004; Ricouer, 1976).

A naïve reading is the first step in the analysis phase, followed by a structural analysis and a comprehensive understanding. The naïve reading involved the first author (AS) reading through the text several times to become moved by it and to gain an initial understanding of the whole. The first naïve understanding was then written down to form a coherent text (Lindseth & Norberg, 2004). The structural analysis was the second step in the analysis process. The transcribed VIO was divided into units of meaning (Table I), followed by a read-through and a reflection against the background of the naïve understanding, all with the aim of the study in mind. The meaning units were then condensed and abstracted into sub-themes, themes and, finally, a main theme. The naïve understanding was validated against the structural analysis and also rewritten once. The text that developed through the analytical process was translated into English upon completion of the analysis. The sub-themes, themes, and main theme, together with the naïve understanding and the authors’ pre-understanding, were reflected on using suitable literature to form a comprehensive understanding (Lindseth & Norberg, 2004). All authors were involved in the analysis process, and the manuscript has been reviewed by other research teams during research seminars.

**Table I.** Meaning units and condensation.

Meaning units	Condensation
Oh how nice you are, ohh you are so beautiful yes … smiles. And I have no sweets to give you … Oh no.	The dog is beautiful, but I have no sweets to give him.
The person puts his left hand on the dog and caresses him. Says: yeah, you’re so nice! The dog goes away, the person following him with his eyes. The dog leaves a ball for the dog handler; the dog handler gives the ball to the person. Says: ha ha ha ha (laughs) looks at the dog, extending his left hand to it. Dog handler: you should sit. Dog handler talks to the dog. The person says: ohh yes, how nice you are. Rocking its head sideways. The person says: I cannot sit like that.	Leaning towards the dog, caressing, laughing and looking at the dog. The dog is well behaved when sitting. Smiling and rocking its head.
The person smiles and feels the dog’s paw. Says: yeah ha ha ha (laughs), smiles, trying to say something, it becomes unclear. The person says: I cannot talk … Stops smiling. Dog handler says: yes you can. The person caresses the dog; the dog lifts its head against the person’s hand. The person says: Don’t you like it? Raising her eyebrows. Dog handler: I think he did. The person continues caressing, smiling a little, caressing, watching the dog.	Touches the dog, smiles, says something indistinct. Caresses the dog, wondering if the dog does not like it. Continues caressing, smiling.

### Ethical considerations

A therapy dog team was established at the nursing home, and the first author was able to join this team while they visited people with AD. Approval for the study was given by the Regional Board of Research Ethics (2010/220-31/1). The participants were all frail owing to moderate to severe AD, and their next of kin were contacted and informed both orally and in writing about the project, and they then signed a proxy consent (Karlawish et al., 2008). The information given was that those with AD would be able to receive therapy dog visits without being part of the research project. The first author collected the data (films) of the participants interacting with the therapy dog and looked for signs that might indicate any discomfort on their part; however, there were no such signs. The VIOs were coded and stored in a safe cabinet to which only the authors had access.

## Results

The findings will be presented with the naïve understanding first, followed by the main theme and each theme with its sub-themes. According to the method of phenomenological hermeneutics, the results are written from the position and lived experience of the person with AD encountering a therapy dog.

### Naïve understanding

The presence of the dog brings joy and closeness for that moment, and also results in a sense of appreciation and moment of happiness in daily life. The dog takes on a value that becomes apparent when one is sharing closeness and warmth, and when talking tenderly to the dog, using skills and knowledge, and taking responsibility for the dog by making the right decisions as to how the dog should be taken care of. Affection through the creation of a safe, warm environment for the dog is achieved through conversation with and about the dog. Caring is about being able to express one’s wish to be important to and needed by the therapy dog, which means that one has done one’s best while being aware of one’s limitations when it comes to caring for the dog.

Caring for the dog is making responsible decisions about what is best for the dog; for example, providing care by being close to the dog, giving it one’s attention, wanting to be useful to the dog, and saying that one always wants to be at the dog’s side as a friend.

### Structural analysis

The structural analysis resulted in one main theme, two themes, and four sub-themes (Table II). In the results, the participants have been given fictitious names.

**Table II.** Sub-themes, themes and main theme that emerged from the text.

Sub-theme	Theme	Main theme
Being affectionate towards the dog Being concerned for the dog Becoming significant for the dog in the moment Distancing oneself from the dog in the moment	Letting one’s feelings lead the way with the dog’s best interests in mind Being close and at a distance	Using one’s own resources and abilities as a human being

#### Main theme: Using one’s own resources and abilities as a human being

Using hidden resources and abilities, one may become the person one once was before cognitive decline, and also distance oneself from the symptoms of the disease during the time spent with the dog. Becoming an autonomous person who shows authority and decides what is best for the dog. Showing one’s inner self, the person one may have been before the symptoms of cognitive decline concealed one’s previous abilities.

#### Theme I: Letting one’s feelings lead the way with the dog’s best interests in mind

Caring meant ensuring security and safety for the dog in different situations by giving love and tenderness; using skills based on memories from life and making sure the dog is safe in the situation at that moment, based on one’s own experiences and beliefs.

***Being affectionate towards the dog*** means providing care by understanding, respecting, and adapting oneself to the dog and the situation, as well as treating it as a precious living creature.

Mrs Anderson: [Sits up and looks at the dog.] Well, you are so cute … Well you are so beautiful. [She bends down towards the dog: *You are so beautiful*. You are so beautiful. [Takes a sweet from a plastic bag and gives one to the dog.] You are so beautiful. [The dog eats the sweet.] Was it good? [Bends down towards the dog and looks at him.] Did you like the sweet? [Still looking at the dog. The dog comes forward and puts it head in Mrs Anderson’s lap. She leans against the dog]. Well you are so beautiful ….

When the dog is close, one acknowledges its presence by showing admiration for it and using calm words like “Yes, you are so good”. In the interaction, one provides care for the dog by appreciating it for being clever and smart when it obeys commands. The dog’s closeness makes one realize that the dog appreciates affection when being stroked since it stays close and falls asleep in one’s presence.

Becoming affectionate towards the dog by putting the dog’s needs before one’s own at that moment. When one is providing care for the dog, the symptoms of one’s disease are less obvious at that moment, and one recalls life memories as a result of protecting the dog and treating it as a fragile creature. Often temporary presence of mind occurs when one is caring for the dog, which also reveals an understanding that one cannot take care of the dog in one’s present condition. Instead, there is an understanding about how the dog should be taken care of, as well as how important the dog is.

One provides care by being responsible and by protecting and expressing worry for the dog about things in the close vicinity that can harm it. At the same time, one is acknowledging the importance of the dog—that it is a vulnerable and beloved friend that one would like to have visit again.

***Being concerned for the dog*** by protecting it, such as putting one’s arms around the dog as a shield when realizing that certain situations may be threatening to the dog; providing care by observing and commenting on any health problems in order to be considerate; showing concern for the dog by understanding that it is not behaving as before—realizing perhaps that something is wrong with the dog.

Mr Edgar: Are you limping, my friend? [He looks at the dog.] Dog handler (DH): Yeah, he has a bit of pain in his front end. Mr Edgar: OK …. [He follows the dog with his eyes. His voice becomes somewhat quieter and darker, and his expression becomes serious.] DH: Do you think it will pass? Mr Edgar: Yes, I hope so … DH: yes … I think so. Mr Edgar: Yeah, let’s hope so. DH: He gets medicine for his limping. Mr Edgar: Ooh well. DH: So, so the vet thinks so. Mr Edgar: Yes, yes it appears that he has problems when he walks … Oh, oh, oh poor little chap, yes, yes.

Caring for the dog by exhibiting cautious behaviour and by trying to solve any problems with the dog’s best interests in mind. The focus moves from joy and closeness to a serious moment when the dog is not feeling well, and one seeks support and confirmation from the handler to understand how to help with the problem.

#### Theme II: Being close and at a distance

Caring for the dog meant creating a special relationship with the dog, such as being quiet and close together, understanding each other; at the same time, moving away when the dog comes too close.

***Becoming significant for the dog*** *for the moment* meant caring for the dog by reacting to and approving of the dog’s behaviour in words to the dog and in play while having the dog’s attention; understanding that one is important for the dog’s well-being in that shared moment with the dog.

Mrs Carlson: Hey you, hey you, little doggy! Hey you, little doggy! [Smiling, she Yes, are you coming to me, are you coming to me …? Well, well I do not have any sweets you see … [Laughing, looks at the dog and looks at the dog handler.] *… ha*… ha [laughs]. [Strokes the dog, smiling.] He wants more pats ….

Becoming significant for the dog meant silently being able to acknowledge the dog by being close to and watching the dog, and attending to the dog’s needs and adapting to the dog; being useful by providing a sense of security to the dog, and at the same time doing something enjoyable. Quietly caring for the dog meant managing to play a game repeatedly as a sign of understanding of the dog and the situation without any verbal commands or control from the handler.

***Distancing oneself from the dog at that moment*** to protect one’s integrity by showing authority and setting limits for both the dog and oneself.

Mrs Daniels: Yeah, ha [laughs], you are so beautiful, yesss. [She caresses and talks to the dog.] I do not … not there, there. [She bends her head back when the dog tries to lick her face.] Soo, yes so beautiful, yes, I do not want you in my face. Noooo. [The dog turns around.]

Being emotional, and stating to the dog that one wishes to be left alone, and instead observing it quietly from a distance, without any further explanation.

## Discussion

### Comprehensive understanding and reflections

The people with AD in this study acted affectionately and confidently while demonstrating their ability to lead, decide, and act in ways that they thought benefited the dog. In Parse’s (1992) theory “Human Becoming”, to be human is the equal right of everyone regardless of background, and the person freely chooses meaning and value in a situation by involving himself or herself. To experience good health, there needs to be an equilibrium between mental, social, and spiritual well-being; this in turn creates equilibrium of body, mind, and spirit (Parse, 1992). The participants caring for the therapy dog demonstrated moments that were full of life and confidence based on their resources and abilities to make decisions about the dog in a given situation. This required a sense of equilibrium of body, mind, and spirit in the situation when the dog was in the room. For a person to regain their health when living with a disease, they need to find personal meaning in a situation and to piece together what is real and what brings meaning to them (Parse, 1992, 1995).

For the participants in this study, the dog may have helped them to find personal meaning through its bodily proximity which, in turn, may have helped to connect them with their inner life world with memories of caring for their dogs in days gone by. Provided care is shown through love and responsibility and by being both close and at a distance, the individual appears to be able to act like a healthy human being at that moment with the dog, with the symptoms of the disease, perhaps, being put aside for the moment.

The findings show the participants in the present study to be calm. They expressed sounds of contentment and almost fell asleep when caressing and being close to the dog. Skovdahl, Sörlie, and Kihlgren (2007) found that when people with dementia in a nursing home received tactile stimulation to their hands and arms from caregivers, they relaxed and fell asleep. This, in turn, reduced symptoms such as aggression and increased their sense of well-being, and they became easier to interact with (Skovdahl et al., 2007). The dog’s coat may have functioned as tactile stimulation for those in this study. The act of providing physical care and interacting with the therapy dog may be closely related to receiving tactile stimulation from a caregiver, as described by Skovdahl et al. (2007). Levels of oxytocin increase when people have physical contact with a dog for 5–24 min which, in turn, reduces stress and pain and results in lower blood pressure (Beetz, Uvnäs-Moberg, Julius, & Kotrschal, 2012). It is possible that the level of oxytocin increases in the person with AD during the time spent caressing the dog.

These calm moments with the dog may also reveal memories from the person’s past (Swall et al., 2014): memories of dogs and memories of their interactions with dogs. These memories may also, albeit temporarily, result in the person with AD behaving like a confident, healthy person. The person may manage to be one “person” on the ward when living with the impending conditions of the symptoms of dementia, which may, in turn, create space to be one “kind of person”, where the “true” self may not be the same as before the onset of cognitive decline.

People with moderate AD have been shown to strengthen their sense of self, their “I”, in relation to the world and to others when interacting with each other in controlled group conversations (Hedman et al., 2014). In this study, the people appeared to become empowered, more secure, and more confident, and to lead the way and make decisions about what was best for the dog, while still understanding that their own limitations might affect the dog. The findings show that caring for the dog makes old people who are experiencing cognitive decline more outgoing, social, and interested in and focused on the dog and its needs, while they also become more prepared to express their sense of self (Hedman et al., 2012, 2014). It appears that the person emerges from the shell created by dementia and may show a fragment of his or her “person” as a result of being empowered in the dog’s presence. Together with the dog, they possibly show their innermost self. They liven up and demonstrate the abilities and resources of a whole human being, and know that they are capable of taking care of the dog using their current faculties.

Revealing a sense of self through communion with others in friendship, dialogue, caring, and togetherness has been shown to be important (Hedman, 2014). People’s inner life and sense of self may come to the fore when they are caring for a friend, the dog, and they may act as if their cognitive decline is not limiting their resources or abilities, thus creating a sense of well-being and optimal health as well as equilibrium of body, mind, and spirit (Parse, 1995).

Episodes of lucidity are moments of temporary presence of mind in people with AD and other dementias (Normann et al., 1998). These moments often occur when the person is cared for in a person-centred way (Edvardsson et al., 2014, 2008; McCormack & McCane, 2010). The dog’s presence seemed to create episodes of lucidity which, in turn, evoked memories and feelings in the individuals, enabling them to show how much the dog meant to them.

### Methodological considerations

This study aimed to increase understanding in terms of the meaning of care for people with AD in their encounter with a therapy dog. AD causes symptoms such as amnesia and aphasia, and therefore it was important to videotape sessions so that the researcher could capture both verbal and non-verbal expressions while the participants interacted with the therapy dog. Because of speech difficulties for most of those participating and because the wish was to produce films with rich verbal and non-verbal data, VIOs seemed to be the best option.

Older people with AD can find it intimidating to be filmed; therefore, the first author showed herself in front of the camera to demonstrate that there was nothing to be frightened of in terms of the camera or the author herself. It is possible that directing the focus from the dog to the first author may have influenced the data; however, most of the time the person looked only at the dog. Any discomfort on the part of the participant was duly observed; however, no such discomfort was noted.

Using phenomenological hermeneutics (Lindseth & Norberg, 2004) involves an ongoing movement between the parts and the whole in the text, and between explanation and understanding (Ricoeur, 1976). The analysis that was required to reach the meaning of the lived experience was not a linear process; instead, it moved back and forth to validate the naïve reading with the structural analysis, and with the aim of the study in mind, a deeper understanding of the phenomenon was attained, as was the way in which it showed itself in front of the text (Lindseth & Norberg, 2004).

It was important in the VIOs to capture every moment that the participants and the dog were together. Five people with AD participated in the study, and 50 films totalling about 25 h of recorded time were transcribed. The richness and variation of the data can be deemed satisfactory: quality is more important than quantity when it comes to examinations of the life world (Dahlberg, Dahlberg, & Nyström, 2008). The authors’ pre-understanding needed to be checked during the structural analysis to maintain truthfulness to the data. This was achieved by all authors through ongoing commentary on the text and by other research teams contributing during seminars. Through the comprehensive understanding, the results illuminated deeper meanings of the lived experience of older people with AD caring for a therapy dog, which demonstrated the impact the dog may have had on the participants. The results may also have presented a platform for the further use of trained therapy dogs in the care of older people with AD and other dementias. Meanings of the lived experience for older people with AD in their encounter with a therapy dog have been disclosed (Ricouer, 1998), but the text never has just one meaning or one “truth”. The present study shows one interpretation of the text, but other interpretations are possible (Lindseth & Norberg, 2004).

## Conclusions

The present study contributes to knowledge about older people with AD caring for a therapy dog. AAT with a therapy dog is said to affect people with dementia by minimizing agitation and anxiety-induced behaviours such as apathy and aggression, while improving their quality of life and their social interaction with others. In this study, other behaviours such as empathy and affection that were demonstrated through expressions of feelings of joy and tenderness were noted. These moments possibly gave them momentary empowerment despite the symptoms of the disease, and may have given rise to a sense of being important, needed, and meaningful to the dog in its presence. The study may be seen as a contribution specifically to research into caring for older people with AD who interact with a therapy dog, and more generally into person-centred care for people with dementia. Animals have been living close to humans for many years, for example, both as farm animals and as companions. Therapy dog visits could be used in different parts of the world and in different cultures under controlled conditions that adopt a person-centred approach.

## Implication for practice

The study contributes to knowledge about AAT with a therapy dog and its different possible applications in the care of people with dementia. Furthermore, AAT with a therapy dog may contribute to a person’s sense of well-being and calm, and may also give meaning to the day for those with dementia. However, there needs to be further research in this area to fully understand the benefits of using AAT with a therapy dog in the care of people with dementia. All authors have contributed differently in the article. However, the first author (AS) collected the data and was leading the analysis process along with the last author (IF). However, all authors have been involved in all parts of the article.
